# Supplementary material for: Mechanisms governing the pioneering and redistribution capabilities of the non-classical pioneer PU.1
Source: Nat Commun. 2020 Jan 21;11:402. doi: 10.1038/s41467-019-13960-2 (PMC6972792; doi:10.1038/s41467-019-13960-2)
Supplement: Supplementary file 7 — Source data [file 41467_2019_13960_MOESM7_ESM.zip › Source_Data/Figure5/Figure5A_MotifScanOutput/homerResults/motif5.similar.html]

motif5

## Information for motif5

T
C
G
A
C
G
A
T
A
T
C
G
C
G
T
A
T
A
G
C
C
G
A
T
T
G
A
C
C
G
T
A
A
C
G
T
G
T
C
A
A
G
C
T
  
Reverse Opposite:  

T
C
G
A
C
A
G
T
T
G
C
A
A
C
G
T
A
C
T
G
C
G
T
A
A
T
C
G
G
C
A
T
T
A
G
C
C
G
T
A
A
G
C
T
  

|  |  |
| --- | --- |
| p-value: | 1e-116 |
| log p-value: | -2.674e+02 |
| Information Content per bp: | 1.665 |
| Number of Target Sequences with motif | 565.0 |
| Percentage of Target Sequences with motif | 18.73% |
| Number of Background Sequences with motif | 2713.6 |
| Percentage of Background Sequences with motif | 5.95% |
| Average Position of motif in Targets | 208.9 +/- 141.2bp |
| Average Position of motif in Background | 209.6 +/- 136.9bp |
| Strand Bias (log2 ratio + to - strand density) | 0.1 |
| Multiplicity (# of sites on avg that occur together) | 1.13 |
| Motif File: | file (matrix) reverse opposite |

### Similar de novo motifs found

|  |  |  |  |  |  |  |  |
| --- | --- | --- | --- | --- | --- | --- | --- |
| Rank | Match Score | Redundant Motif | P-value | log P-value | % of Targets | % of Background | Motif file |
| 1 | 0.971 | A C T G T G C A C G A T A C T G G C T A A T C G C G A T T A G C C G T A A G C T | 1e-109 | -253.094135 | 22.58% | 8.55% | motif file (matrix) |
| 2 | 0.982 | C T A G A T C G C G A T T C A G A C G T A C T G C G T A T A C G C G A T G T A C C G T A A G C T G T C A C A G T | 1e-108 | -250.879247 | 14.59% | 3.93% | motif file (matrix) |
| 3 | 0.979 | T G C A C G A T A C T G C G T A T A G C A C G T A G T C G T C A A C G T T G C A T A C G G C T A | 1e-106 | -244.353368 | 13.53% | 3.48% | motif file (matrix) |
| 4 | 0.965 | T C A G A C G T A C T G C G T A A T C G A C G T A G T C C G T A A C T G G T A C C G A T T G C A T A C G | 1e-105 | -242.105953 | 15.98% | 4.80% | motif file (matrix) |
| 5 | 0.965 | A C G T A C T G C G T A T A C G A C G T A G T C C G T A A G C T | 1e-104 | -241.202414 | 23.71% | 9.54% | motif file (matrix) |
| 6 | 0.969 | G C A T C A T G G C T A A T C G C A G T A T G C C G T A A C G T G T C A | 1e-103 | -237.253137 | 23.41% | 9.43% | motif file (matrix) |
| 7 | 0.934 | A C G T A C T G C G T A A C T G A C G T A G T C C G T A | 1e-94 | -217.775668 | 19.69% | 7.45% | motif file (matrix) |
| 8 | 0.831 | T C G A C G A T A T C G C G T A A T C G C G A T T G A C G C T A C A G T A T C G G A T C G A C T A C G T | 1e-43 | -101.134370 | 8.22% | 2.81% | motif file (matrix) |
